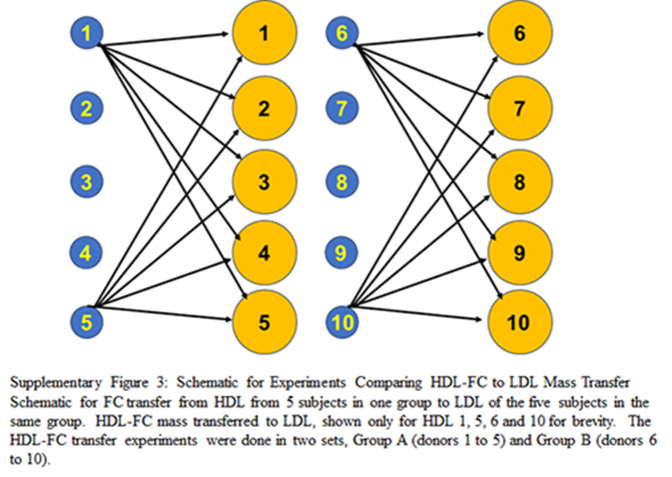# HDL-free cholesterol influx into macrophages and transfer to LDL correlate with HDL-free cholesterol content

**DOI:** 10.1016/j.jlr.2024.100707

**Published:** 2024-11-19

**Authors:** Dedipya Yelamanchili, Baiba K. Gillard, Antonio M. Gotto, Miguel Caínzos Achirica, Khurram Nasir, Alan T. Remaley, Corina Rosales, Henry J. Pownall

**Affiliations:** 1 Department of Medicine, Houston Methodist, Houston, TX, USA; 2 Department of Medicine, Weill Cornell Medicine, New York, NY, USA; 3 Hospital del Mar Research Institute, Barcelona, Spain; 4 Department of Cardiology and Houston Methodist DeBakey Heart & Vascular Center, Houston Methodist, Houston, TX, USA; 5 Lipoprotein Metabolism Laboratory, Translational Vascular Medicine Branch, National Heart, Lung, and Blood Institute, National Institutes of Health, Bethesda, MD, USA

**Keywords:** cholesterol/trafficking, LDL, Lipoprotein/Kinetics, Cholesterol/Metabolism, Lipids, HDL function, assay reproducibility

## Abstract

High-density lipoprotein (HDL)-free cholesterol (FC) transfers to other lipoproteins and cells, the former by a spontaneous mechanism and the latter by both spontaneous and receptor-mediated mechanisms. Macrophages are an important cell type in all stages of atherosclerotic cardiovascular disease (ASCVD), and the magnitude of FC efflux from macrophages to HDL, a metric of HDL function, inversely associated with several metrics of ASCVD. Very high plasma HDL concentrations are associated with increased all-cause and ASCVD mortality, suggesting that the reverse process, FC influx from HDL into macrophages, is atherogenic. We hypothesize that HDL-FC is a metric of dysfunctional HDL, and when combined with HDL particle number (HDL-P), is an ASCVD risk factor. The magnitude of FC influx from HDL to macrophages is expected to be a function of HDL-P and HDL-FC content. Here we show that plasma HDL-FC content varies 2-fold among normolipidemic human subjects and linearly correlates with low-density lipoprotein (LDL)-FC content. The influx of HDL-FC into macrophages and transfer to LDL increase linearly with HDL-FC. As expected, the influx of HDL-FC into macrophages and the transfer to LDL are positively correlated. These data support the hypothesis that high HDL FC content is a marker for dysfunctional HDL, resulting in greater influx into macrophages and greater HDL-FC transfer to LDL. HDL-FC transfer to LDL is a valid surrogate for influx into macrophages. This study of HDL composition and function of normolipidemic subjects provides the basis for further investigation and establishment of HDL-FC content as an ASCVD risk factor.

Numerous (1, 2) studies have revealed that plasma concentrations of LDL-C and HDL-C are, respectively, positive and negative risk factors for atherosclerotic cardiovascular disease (ASCVD). The mechanistic link between elevated plasma LDL-C has been established through many studies including those of hypercholesterolemic patients with mutations in the LDL-receptor (3) and those related to the development of the statin class of drugs that lower plasma LDL-C and with it ASCVD incidence (4). Mechanistic connections between reduced HDL and increased ASCVD and therapies that target HDL concentrations have been more elusive and contradictory. Whereas lowering plasma LDL-C reduces ASCVD events, therapies that raise HDL-C concentrations have failed to do so, especially on a background of statin treatment (5). These therapies included niacin, fibrates, and especially inhibitors of cholesteryl ester transfer protein, which raise HDL-C concentrations. Cholesteryl ester transfer protein (CETP) inhibitors were expected to be powerful anti-ASCVD agents because some increased plasma HDL-C concentrations by as much as 100%; however, their effects on ASCVD events were either nil or unimpressive (6, 7, 8, 9), and associated with reduced non-HDL-C rather than increased HDL-C (10, 11). Also, normolipdemic and hypertriglyceridemic Japanese-American men with higher than normal plasma HDL-C concentrations due to CETP deficiency exhibited increased ASCVD risk (12, 13). Subsequent studies shifted to models of HDL function in which the free cholesterol (FC) burden of macrophages, an important cell type in the pathogenesis of ASCVD, was reduced by FC transfer to HDL, a process known as FC efflux. The seminal study found that macrophage FC efflux inversely correlated with both carotid artery intima-media thickness and the likelihood of ASCVD, independent of the HDL-C concentrations. (14) This finding was affirmed in other studies including population-based cohorts (15, 16).

Paradoxically, several recent studies revealed that very high plasma HDL concentrations are associated with increased all-cause (17, 18, 19, 20) and ASCVD mortality (21). Although the underlying causes for these associations are not known, some clues emerge from studies of the HDL receptor-deficient (Scarb1^−/−^) mouse, a pre-clinical model of human ASCVD in the context of very high plasma HDL-C concentrations, that is, a high particle number (HDL-P) and FC-rich HDL so that the plasma HDL-FC concentration is 7–8 times that of wild-type mice (22). The combination of a high HDL-P and FC-rich HDL gives rise to a state of high HDL-FC bioavailability index (BI) (16) defined by

(1) HDL-FCBI = HDL-P × HDL-mol% FC

where HDL-P is the HDL particle number and

(2) HDL-mol% FC = 100 × mol_FC_/(mol_FC_ + mol_PL_)

where mol_FC_ and mol_PL_ are the respective numbers of moles of FC and PL. In the context of Equation 2, FC is the solute, and PL is the solvent, and by analogy with Raoult’s law, the lower the solute to solvent ratio, the lower the vapor pressure of the solute or in our system, lower FC bioavailability. According to Equation 1 and published HDL compositional data (22, 23), HDL-FCBI of 73 for Scarb1^−/−^ mice is greater than the values of 12 for normolipidemic human HDL and 8.3 for WT mouse HDL (16).

The high HDL-FCBI observed in Scarb1^−/−^ mice is associated with abnormal erythrocyte structure, (24) female infertility (25), FC-enrichment of many tissues (22), and ASCVD (25). In Scarb1^−/−^ mice, reduction of the high HDL-FCBI with probucol or bacterial serum opacity factor normalizes erythrocyte structure, (26) rescues female fertility (27, 28), and prevents ASCVD (29). FC flux is bidirectional. In our earlier work (30) we studied the effects of HDL FC content on FC flux between HDL and human skin fibroblasts. At low HDL-FC content, HDL was an acceptor of FC from the cells but above 15 mol%FC, HDL was an FC donor to cells rather than an acceptor. Moreover, FC influx from the FC-rich HDL of Scarb1^−/−^ mice to macrophages is higher (+300%) than that of wild-type mice with little difference in FC efflux (22). Thus, the HDL-FC content determined the direction of the FC movement to both fibroblasts and macrophage cells. This has led to the hypothesis for the current study that at high HDL-FC content HDL becomes a donor of FC to LDL and cells, thereby providing a hypothetical mechanistic link between a high HDL-FCBI and atherogenesis (22).

The relationship between high HDL-FCBI and atherogenesis has never been tested in humans. Given that the cell and pre-clinical data show that a high HDL-FCBI is associated with ASCVD, we have begun a four-year clinical trial, the Houston Heart Study, to test in humans the hypothesis that a high HDL-FCBI is associated with dysfunctional HDL, as measured by increased HDL-FC transfer to macrophages and excess ASCVD. Prior to the initiation of assays on clinical samples, we validated our assays for lipoprotein lipid composition and HDL function by determining within-day and day-to-day reproducibility. Here we describe and validate robust assays for HDL composition, including one component of HDL-FCBI, i.e., HDL-mol% FC (Equation 2), and its correlation with the functional assays of HDL-FC influx into macrophages and transfer to LDL using autologous HDL from ten normolipidemic subjects. Whereas ASCVD has been correlated with impaired FC efflux in numerous studies (Reviewed) (16), there have been few studies on the role of HDL-FC influx in ASCVD.

## Materials and Methods

### Materials

Plasma (240 ml) was obtained from two pools of five volunteers who donated blood at the Houston Methodist Blood Donor Center, approved for de-identified research use by the Houston Methodist Research Institute Institutional Review Board (Pro00012908). HDL and LDL were isolated from the plasma by sequential flotation at densities of 1.006, 1.063, and 1.21 g/ml. Purity was verified by size exclusion chromatography (31). Purified lipoproteins were stored at 4^°^C in Tris-buffered saline (TBS) containing 1 mM EDTA and 1 mM sodium azide. This buffer was also used as the diluent for protein and lipid assays.

Plasma lipid and HDL compositional analyses were determined in triplicate by enzymatic assays for FC, total cholesterol, phospholipid (PL), and triglyceride (TG) (Fuji Film/Wako) and DC-protein assay (BioRad) for protein. Plasma HDL-C concentrations were determined as the total cholesterol concentration of the supernatant remaining after heparin-Mn^+2^ precipitation of non-HDL-C (32). The heparin-Mn+2 reagent was prepared according to Warnick and Albers (33). Cholesterol ester (CE) concentration was calculated as (mg total cholesterol (TC) - mg FC) x 1.6; multiplication by 1.6 accounts for the molecular weight of CE being 1.6 times that of FC, which is what is measured. Reproducibility of the protein and lipid assays was determined on ten HDL and ten LDL samples assayed for each analyte, n = 20. Within-day variability is the average %CV for two size aliquots, each assayed in triplicate (n = 6 for each sample). Day-to-day %CV is the average for two independent assays done on different days by two different research staff members (supplemental Table S1).

### HDL radiolabeling

The radiolabeling protocol has been described previously (34). An ethanolic solution of [^3^H]FC was transferred to filter paper. The solvent was air-evaporated, and the [^3^H]FC was dried onto filter paper transferred to a solution of HDL in TBS, and incubated overnight at 4^°^C. Previous protocols (34) used overnight incubation at 37^°^C, but we found that 4^°^C incubation resulted in good transfer of [^3^H]FC to the lipoprotein, with 50%–90% uptake, but with less lipoprotein aggregation. The filter paper was removed giving HDL-[^3^H]FC. Size exclusion chromatography of the radiolabeled HDL showed that the [^3^H]FC was associated with the HDL. [^3^H] FC-specific activity was based on the HDL-FC concentration and β-counting of an aliquot.

### Equilibrium distribution of [3H]FC between LDL and HDL

Our previous study showed that HDL-[^3^H]FC equilibrates with LDL in less than 30 min (22). For this study, human LDL (1.0 mg protein/ml) and HDL-[^3^H]FC (1.0 mg protein/ml) were co-incubated for 2 h at 37°C after which the LDL was precipitated with heparin-Mn^+2^ reagent as above (32, 33) and aliquots of the supernatants, containing HDL, were β-counted. The mass of HDL-FC transferred was based on HDL-associated β-counts and the [^3^H] FC-specific activity. Within-day reproducibility for the transfer assay was a mean %CV = 2.41 ± 0.35, with the assay done in triplicate or quadruplicate, for a total of 110 independent experiments. Day-to-day reproducibility was calculated as the ratio of two independent transfer assays done 2–6 days apart, as the ratio of dpm transferred to LDL, mean %CV = 1.81 + 1.07, n = 55 independent assays. Reproducibility over time was tested using HDL and LDL samples that had been stored at 4^°^C more than ten weeks: this gave values for pmol FC transferred that were not significantly different from the initial values (*P* = 0.388 and 0.843). (supplemental Table S4).

### HDL-FC influx into macrophages

HDL-FC transfer to macrophages i.e., FC influx, was quantified essentially as described (22). Macrophages (J774A.1, ATCC® TIB-67™), derived from a murine macrophage cell line, were seeded into 12-well plates. [^3^H]FC-labeled HDL (50 μg HDL protein/ml media) was incubated in quadruplicate with macrophages for two hours after which media were removed and the cells washed three times with cold buffer. Cell lipids were twice-extracted with isopropanol and the combined extracts evaporated and β-counted. Residual cell protein was solubilized in one mL NaOH (0.1 M), and the protein was quantified (BioRad DC). The influx was expressed as pmol cell-associated FC/mg cell protein. Within-day reproducibility was determined by 20 independent assays, each done in quadruplicate: within-day %CV, mean ± SD = 4.6% ± 1.8%. Day-to-day variability was determined by repeating the influx assay on four different days for each of the 10 HDL: day-to-day %CV, mean ± SD = 9.4% ± 1.9% (supplemental Table S6).

### Data analysis

Statistical analysis was done using Graph Pad Prism 9 and regression lines were calculated with SigmaPlot 15. All of the transfer assays and HDL compositional analyses were conducted on the same two groups of five participants each.

## Results

### Lipoprotein compositions

**Supplemental Figure S1:**
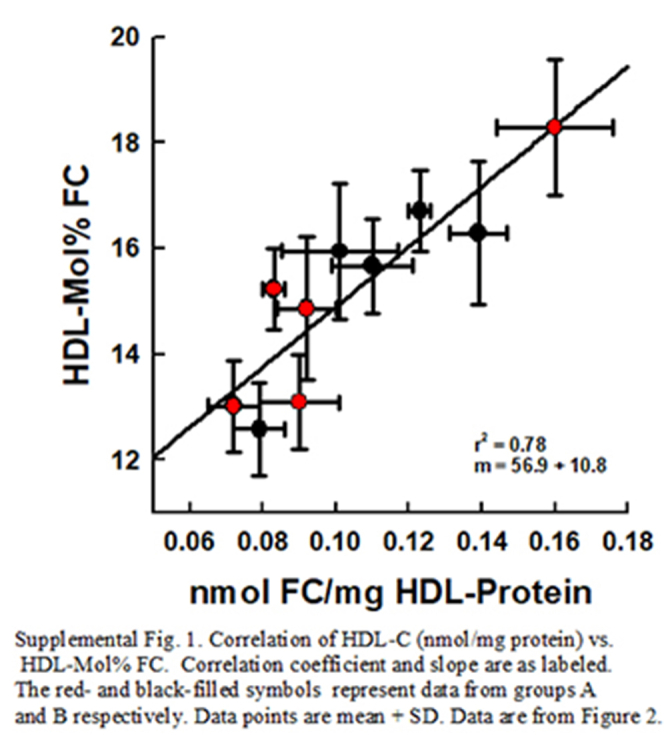


HDL-FC content was expressed in two ways: conventionally, as FC mass/mg HDL protein, and as mol% FC (as described in Equation 2). Given that nearly all FC is partitioned into phospholipids, the essential FC solvent, differences in mol% reflect differences in the cholesterophilicity of each PL pool, which are a function of the identity and abundance of individual PL species (35). For the ten HDL samples assayed here, the nmol FC/mg HDL-protein correlated well with HDL mol% FC (supplemental Fig. S1).

**Fig. 1:**
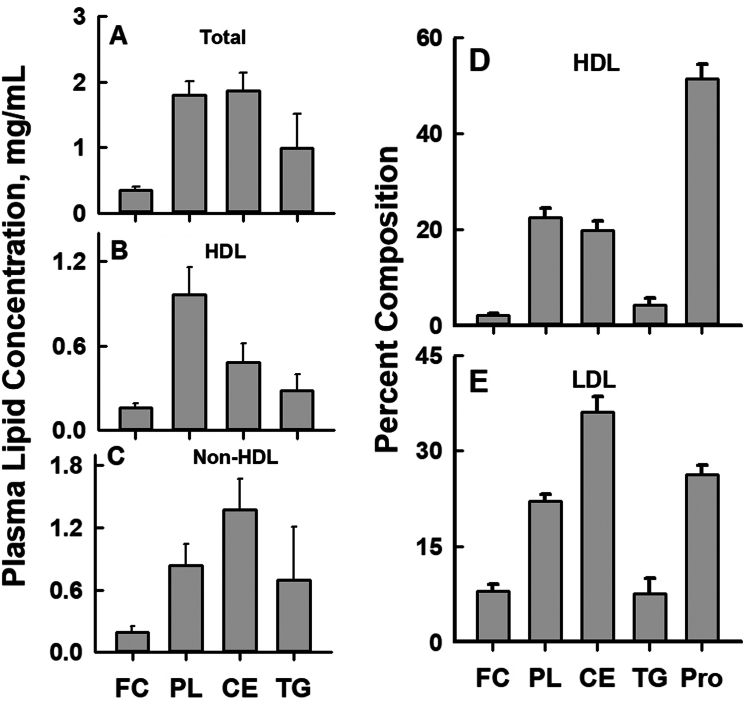
Lipoproteins compositions of ten donors. A–C: Plasma Total-, HDL- and non-HDL-Lipid Concentrations. D and E: Purified HDL and LDL Weight Percent Compositions. Data are mean ± SD.

We first established that our donor plasma was from normolipidemic donors. The plasma and plasma HDL-C lipid concentrations of the ten individual donors within the two groups of five (Group A, donors 1–5 and Group B, donors 6–10) are shown in supplemental Table S2. Where appropriate, graphical data for Groups A and B respectively are shown as red and black symbols. Plasma total cholesterol ranged from 119-177 mg/dl, with a mean of 152 ± 22 mg/dl, and plasma HDL-C ranged from 27 to 61 mg/dl with a mean of 46 ± 11 mg/dl. Plasma, HDL, and non-HDL lipid FC, CE, PL, and TG concentrations were also within the range for normolipidemic subjects (36) (Fig. 1A–C and supplemental Table S2).

**Fig. 2:**
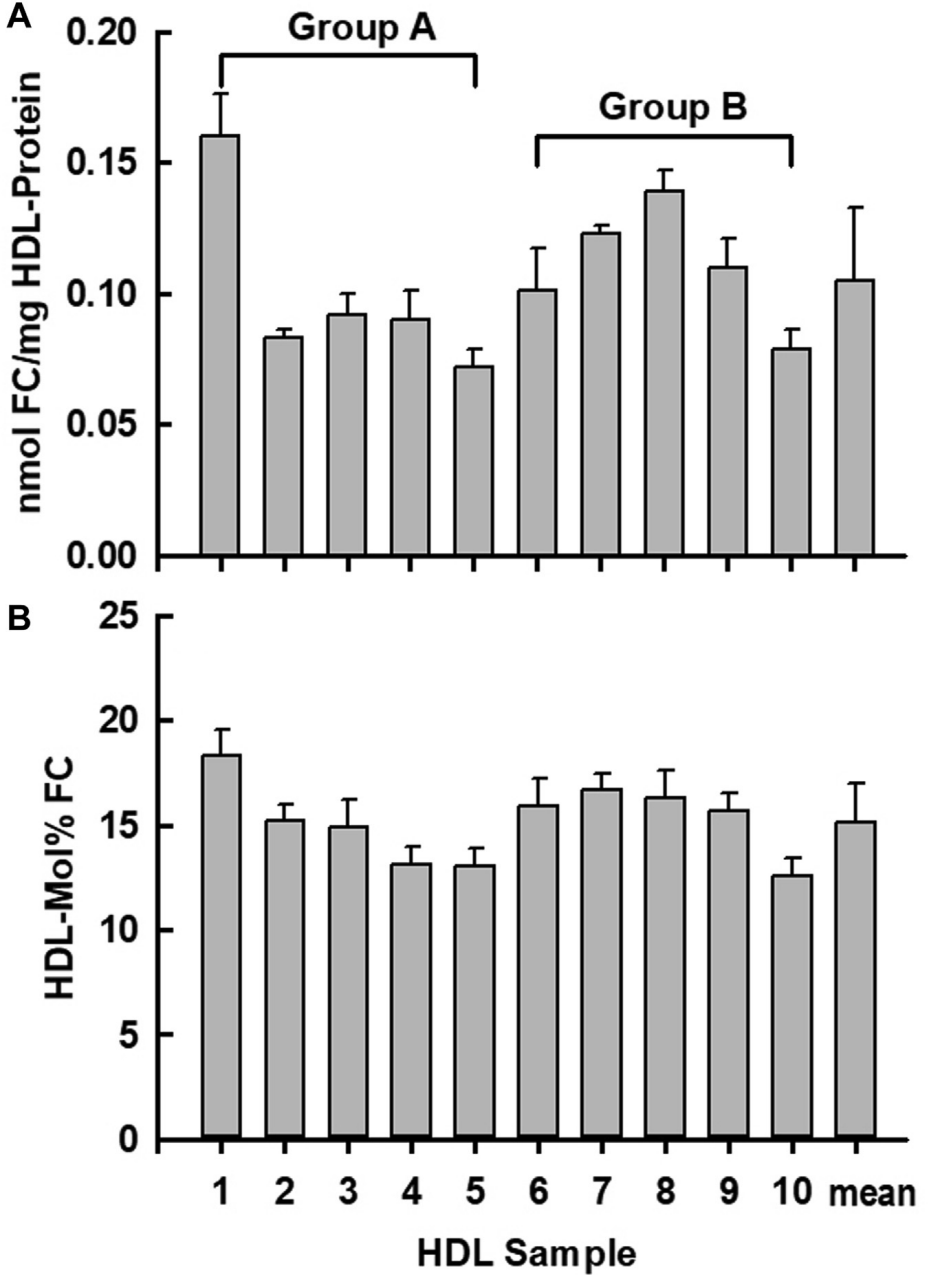
HDL FC concentrations of purified HDL from ten donors in two groups of five, A and B, as labeled. For Group A and B respectively, the ranges of HDL-FC were 0.072–0.16 and 0.079–0.139 nmol FC/mg protein (overall mean = 0.105) or alternatively, 13.02 to 18.29 and 12.58–16.71 mol% FC (overall mean = 15.71 mol% FC). Data are mean ± SD.

HDL and LDL were isolated from each donor plasma and assayed for protein and lipid composition. (supplemental Table S3 and Fig. 1D, E). The HDL-FC contents differed, ranging in molar units from 0.072 to 0.160 nmol FC/mg protein (mean = 0.11 ± 0.03 nmol FC/mg protein; Fig. 2A), which in weight units corresponds to 0.028–0.062 mg FC/mg protein (mean = 0.041 ± 0.011 mg FC/mg protein). The range for HDL-mol% FC was 12.6–18.3 (mean = 15.2 ± 1.8; Fig. 2B). HDL nmol FC/mg HDL-protein were highly correlated with HDL mol% Fc (supplemental Fig. S1.) The HDL and LDL protein and lipid weight percent compositions, which show that HDL was PL-rich whereas LDL was CE-rich, were similar to reported consensus values (36) (Fig. 1D, E). HDL- and LDL-FC contents were respectively 0.105 ± 0.028 and 0.794 ± 0.141 nmol/mg protein, corresponding to 15.2 ± 1.8 and 41.3 ± 3.2 mol% (Fig. 2A, B and supplemental Table S3).

**Fig. 3:**
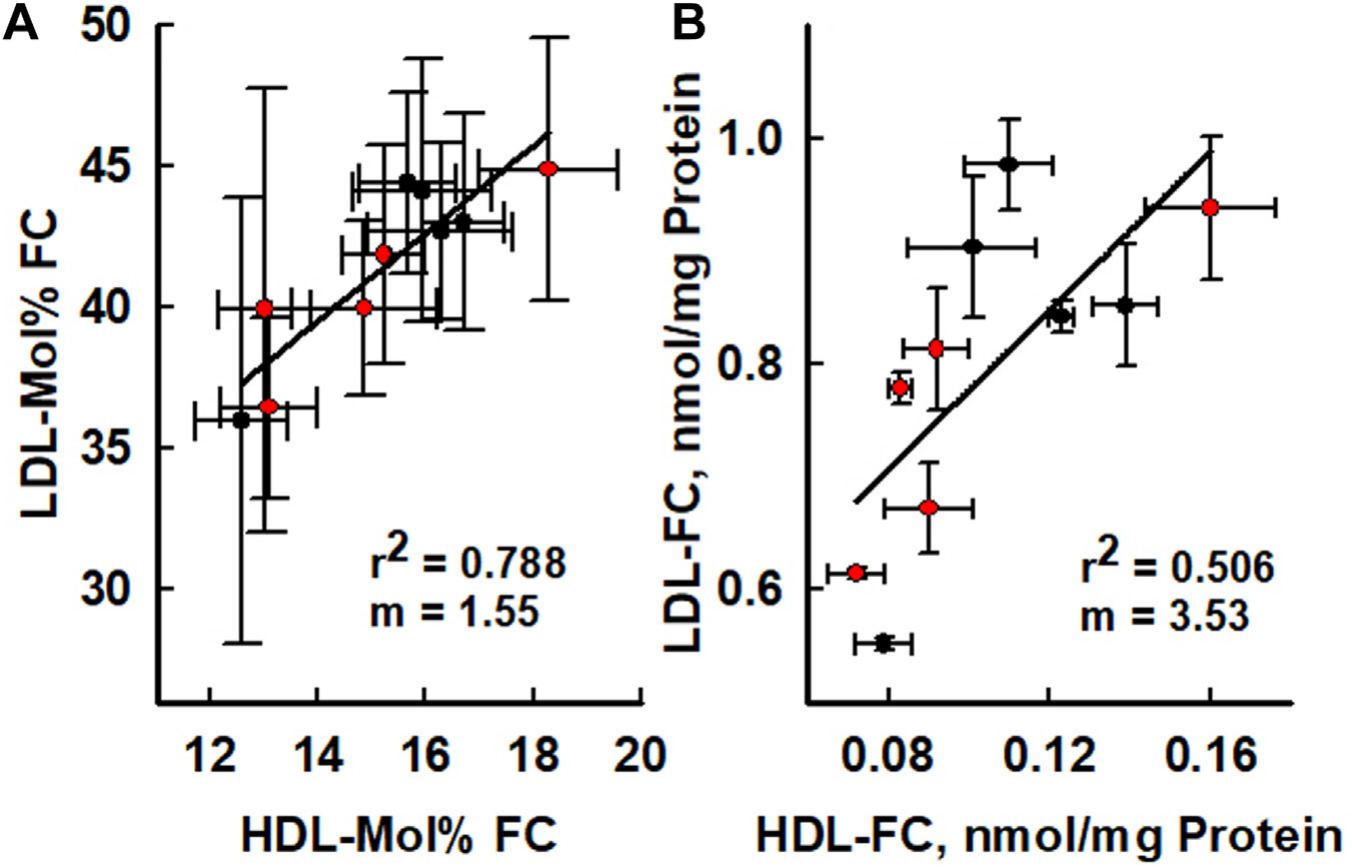
Correlation of HDL- and LDL-FC content according to (A) mol% (range = 12.6–18.3 and 36.0–44.9 mol% respectively) and (B) FC/protein ratio (range = 0.079–0.160 and 0.552–0.938 nmol/mg protein respectively). The red- and black-filled symbols represent data from Groups A and B respectively. Data are mean ± SD.

**Supplemental Figure S2:**
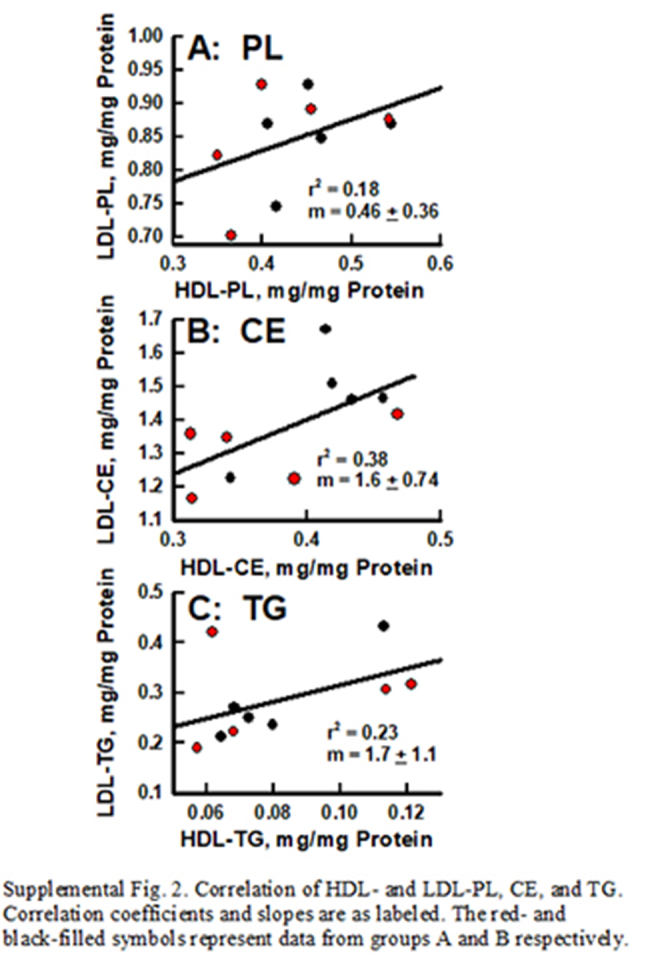


The HDL-FC content of each individual donor correlated well with the LDL-C content for the same individual whether calculated as mol% FC (*r*^2^ = 0.788) or nmol FC/mg protein (*r*^2^ = 0.516; Fig. 3A, B). We also tested whether the content of the other HDL lipids, PL, CE, and TG, HDL, correlated with those of LDL according to their correlation coefficients, slopes, and differences between the highest and the lowest values for each lipid (supplemental Fig. 2A–C). Correlation coefficients for HDL lipids with those of LDL were: *r*^2^ = 0.18 for PL, 0.38 for CE and 0.23 for TG. The corresponding slopes were 0.46, 1.6, and 1.7 and the percent difference between the lowest and highest values for HDL-PL, CE, and TG were +54, +50, and +112% respectively. The corresponding values for HDL-FC versus LDL-FC were higher whether expressed as mol% FC (Equation 2, Fig. 3A; *r*^2^ = 0.79) or nmol FC/mg protein (Fig. 3B; *r*^2^ = 0.51). The respective slopes were 1.55 and 3.53 and the highest HDL-FC value was +122% higher than that of the lowest. Thus, in addition to stronger correlations between HDL-FC and LDL-FC compared to the other lipids, HDL-FC as mol% FC or FC mass/mass HDL protein covered a larger range. According to the slopes of the curves, each 1% change in HDL-mol% FC was associated with an increase of 1.55 LDL-mol% FC, and each 1 nmol increase in HDL-FC/mg protein was associated with a 3.53% increase in LDL-FC nmol/mg protein. As expected from the data of Fig. 3A, B, HDL-mol% FC correlates with nmol FC/mg HDL protein (supplemental Fig. S1).

### HDL-FC transfer to LDL

**Fig. 4:**
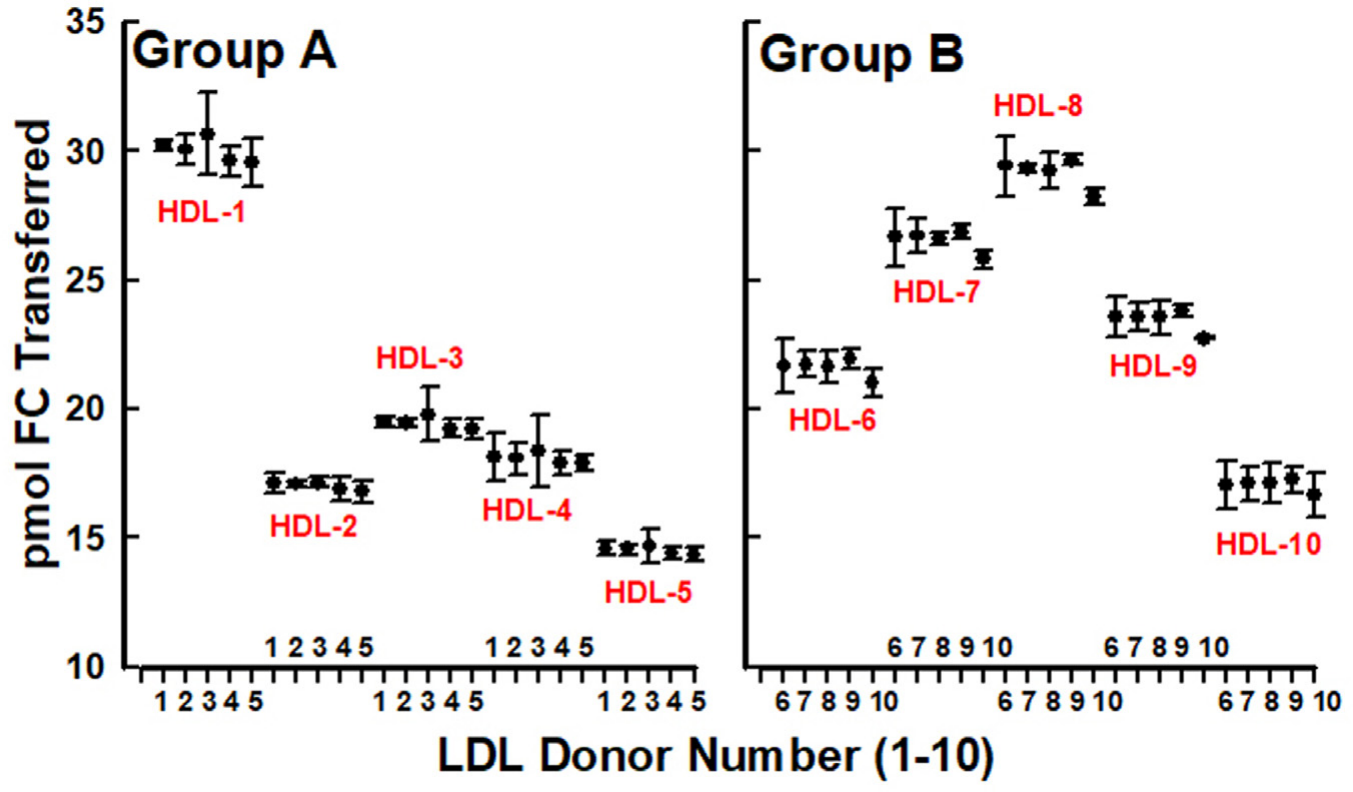
Dynamics of HDL-FC transfer to LDL. HDL and LDL were isolated from two groups of five subjects. After radiolabeling the HDL with [^3^H]FC, the mass of FC transferred from each HDL in each group to each LDL in the same group was determined according to LDL-associated [^3^H]FC. The data show that HDL-[^3^H]FC transfer is independent of the source of LDL but varied by about a factor of two according to the source of HDL, range = 14.5–30 pmol FC transferred/assay (See supplemental Table S5).

The reproducibility of HDL-[^3^H]FC to LDL transfer was determined using HDL and LDL from two groups (A and B) of five donors each. Within each group, we measured FC transfer from each of five HDL samples to each of five LDL samples giving a total of fifty separate triplicate assays (schematic is shown in supplemental Fig. S3). Within-day and day-to-day reproducibility for the HDL-FC to LDL transfer assay, and stability over time are shown in supplemental Table S4. Our data showed that the amounts of FC transferred from the different HDL varied according to participants from 15 pmol to 30 pmol FC (Fig. 4 and supplemental Table S5). However, the magnitudes of FC transfer from a given HDL to each LDL within the five different donors in each group were not different, with %CV for the 5 LDL < 2% (Fig. 4 and Supplemental Table S5). Thus, FC transfer from HDL (the donor) to LDL (the acceptor) is independent of the source of LDL but dependent on the source of donor HDL. This suggests that HDL-FC bioavailability according to HDL-FC to LDL transfer can be determined with good precision with multiple sources of normolipidemic LDL.

### HDL-FC correlates with FC transfer to LDL and influx into macrophages

**Fig. 5:**
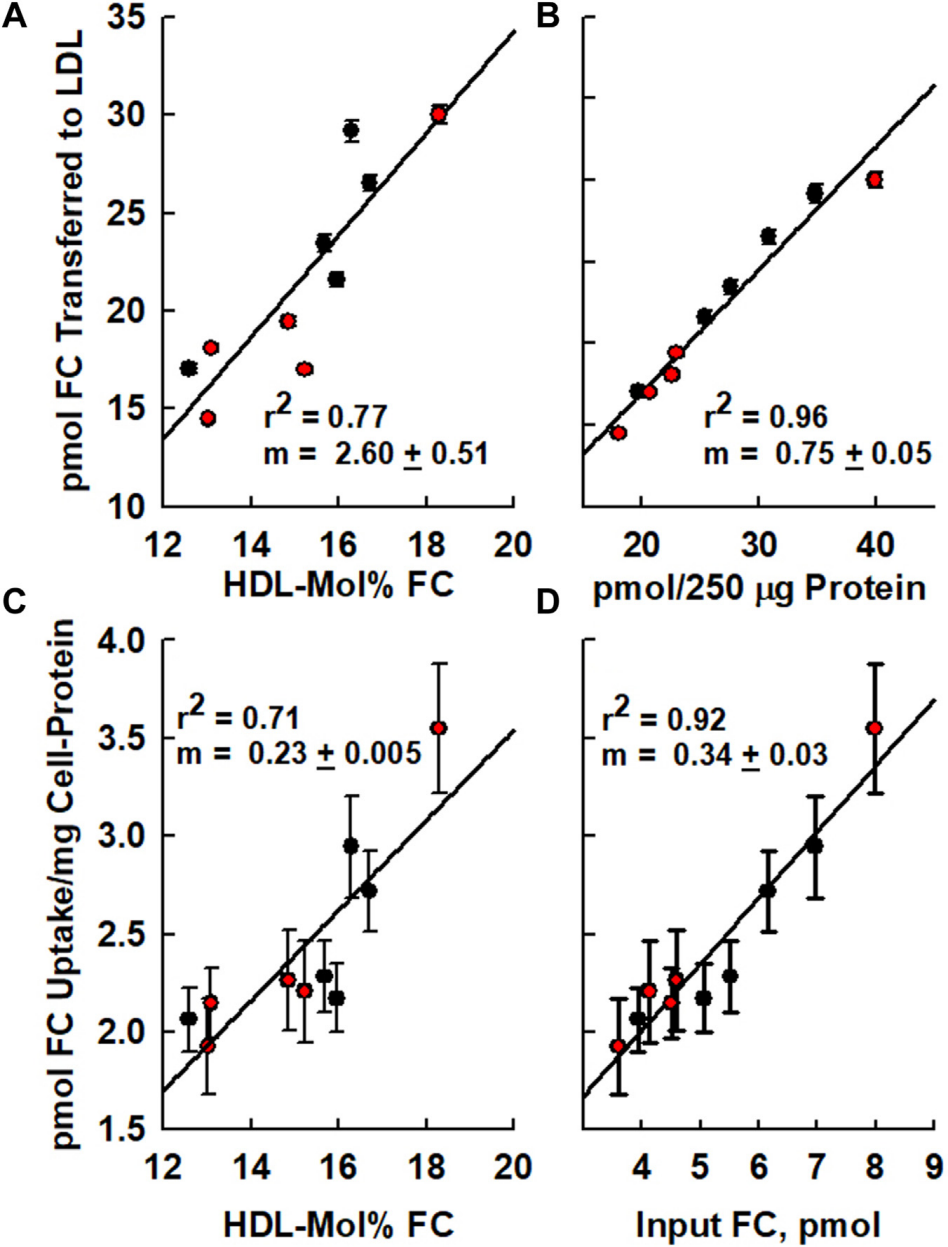
HDL-FC transfer to LDL and influx to macrophages. A and B: HDL-FC Transfer to LDL. C and D: HDL-FC influx into macrophages. Transfer and influx data are expressed according to HDL-mol% FC (A, C) and HDL-FC content (B, D). Data points are mean ± SD.

**Fig. 6:**
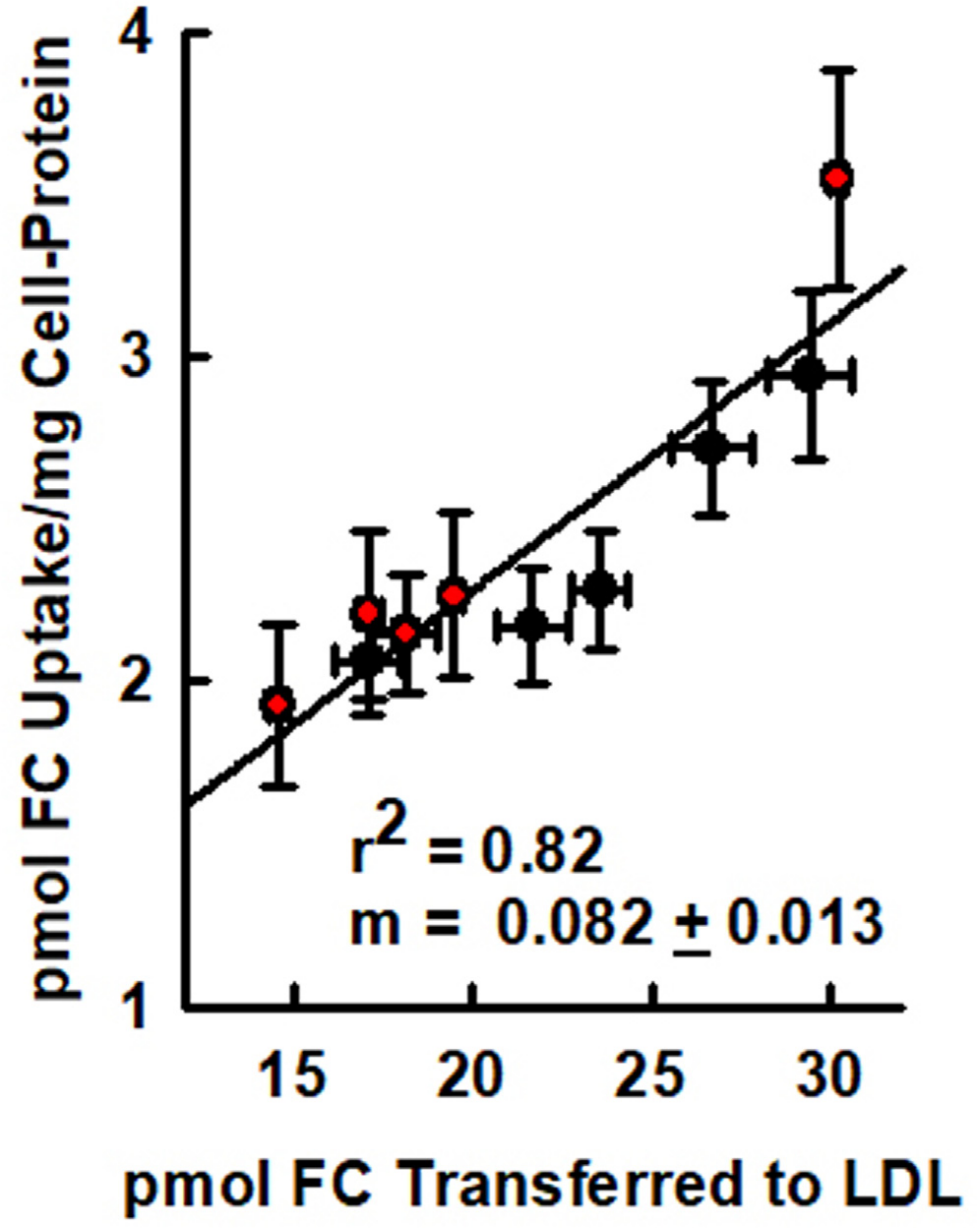
Correlation of HDL-FC transfer to LDL versus HDL-FC influx into macrophages. Data points are mean ± SD.

Given that FC spontaneously transfers among lipoprotein particles (37), we determined the mass of HDL-FC transferred to LDL by each of the ten HDL samples at equilibrium. Whether based on HDL-mol% FC or HDL-FC/mass of HDL-protein, the correlation between HDL-FC transfer to LDL and the initial HDL-FC mass was positive and linear (Fig. 5A, B). Similarly, we quantified the transfer of HDL-FC mass to macrophages. Within-day and day-to-day reproducibility of the HDL-FC influx to J774 macrophages is given in supplemental Table S6. For HDL-FC influx to macrophages, there was a positive linear relationship with respect to HDL-mol% FC and HDL-FC content (Fig. 5C, D). Considering the positive linearity of HDL-FC transfer to both LDL and macrophages, we then compared the two sets of transfer data and, as expected, observed that the transfer of HDL-FC to LDL and the influx into macrophages were also linearly correlated (*r*^2^ = 0.82; Fig. 6).

## Discussion

### Energetics of HDL-FC transfer to LDL

HDL-FC equilibration with LDL is expected to be rapid. Indeed, FC transfer from HDL and LDL occurs with t_1/2_ = 3 and ∼45 min respectively (37). For both transfer to LDL and influx to macrophages, the mass of FC transfer increases with the HDL concentration, and the HDL-FC content is expressed as mol% FC(22, 30) (Fig. 5). Of note, the HDL-mol% FC of most of our subjects, was less than 15 mol%, whereas many patients in the Houston Heart Study have HDL-mol% FC > 15 mol% (Data not shown). Given that the magnitude of macrophage FC efflux to HDL is a metric for ASCVD, in the Houston Heart Study, we hypothesized that the reverse process, HDL-influx into macrophages, is a metric for dysfunctional HDL and a potential predictor of ASCVD under conditions of high HDL-FCBI (Equations (1), (2)) (16). Thus, our goal was to validate reproducible assays for HDL-FC influx into macrophages and transfer to LDL, and to test the hypothesis that HDL-FC influx into macrophages and transfer to LDL are positive functions of HDL-FC content in two groups of five subjects. The former was achieved by measuring the influx of HDL-FC into macrophages. The latter was determined by measuring FC transfer from the HDL of each subject in each group of two groups to the LDL of each participant in the same group.

Despite some variability, particularly for FC content, the lipoprotein compositions and plasma lipid concentrations of all subjects were within the normolipidemic range. Two observations suggest that the affinity of FC for LDL is greater than that for HDL. According to their compositions (Fig. 1D, E and supplemental Table S3), the mean LDL-FC content expressed as weight % FC is higher than that of HDL, 8.0 and 2.1 weight % FC. LDL-mol% FC is also higher than HDL-mol% FC, with mean values of 41.3 versus 15.2 mol% FC. Second, the slopes of the curves of LDL-FC content versus that of HDL, whether expressed as mol% FC (m = 1.55) or FC mass/protein mass (m = 3.53) are greater than 1 (Fig. 3). Given the rapid transfer of FC between HDL and LDL (ie, on the scale of minutes), versus the plasma lifetimes of HDL and LDL (days), the FC distribution between HDL and LDL in plasma is essentially at equilibrium so that an equilibrium constant can be calculated as K = 41.3 mol%/15.2 mol% = 2.72. From this, we calculate the free energy of FC transfer from HDL to LDL as ΔG = -RT∗lnK = 600 cal. The higher affinity of FC for LDL than for HDL explains in part the nearly ten-times slower rate of FC transfer from LDL compared to that from HDL (37).

The range of HDL-lipids was greatest for FC, +122% from lowest to highest. The underlying cause for variation in individual HDL-FC content is not known but could be due to differences in the content of sphingomyelin, the most cholesterophilic of the abundant naturally occurring PL. Although the HDL lipid compositions correlated positively with those of LDL, the correlation was strongest for FC (Fig. 3 and supplemental Fig. S2). The plasma halftimes of LDL and HDL are on the order of 3 and 5 days (38, 39) In contrast, the spontaneous transfer halftimes of HDL- and LDL lipids is shorter, 5 and 45 min respectively (37). Thus, during the long plasma halftimes of HDL and LDL, FC can exchange between the two lipoproteins many times thereby achieving dynamic equilibrium. The poorer correlations of the other HDL-lipids with those of LDL are due to several factors. Having long acyl chains, CE, TG, and PL are highly lipophilic and consequently transfer at slow rates, and transfer depends on inefficient lipid transfer proteins, which support lipid transfer but at rates much slower than that of spontaneous FC transfer. Given that HDL-PL is a substrate for several enzymes, including lipoprotein phospholipase A_2_ and lecithin:cholesterol acyltransferase, (40) its plasma lifetime is greatly reduced (34, 41). Although the hepatic HDL receptor, encoded by the Scarb1 gene, also affects lipoprotein compositions by selective uptake of all plasma lipids (42), this occurs on a slow time scale of hours versus minutes for FC transfer (43). Thus, the much faster rates of FC transfer compared to that of other lipids grounds the expected HDL- and LDL-FC equilibration and support the validity of our HDL-FC to LDL transfer assay.

### HDL-FC to LDL transfer

Longitudinal studies require a reliable assay and durable standards that retain their informative integrity over time. In our case, we needed a source of LDL that would accept HDL-FC with good precision and reproducibility. We had several choices. We could prepare a pool of LDL from multiple donors, frozen or unfrozen, and retrieve aliquots as needed. In so doing, there was concern that both freezing and time would alter LDL structure in a way that alters function, ie, accepting a given amount of HDL-FC with good precision in repeat measures. We could also prepare fresh samples regularly with the expectation that LDL from any normolipidemic subject would function similarly in our FC transfer assays, and we tested this possibility. As hypothesized, we observed that the amount of FC transferred from a given HDL sample was a function of HDL-FC content expressed as mass FC/mg protein or as mol% HDL-FC. Remarkably, the amount of FC transferred from a given HDL to LDL was independent of the source of normolipidemic LDL and in support of our hypothesis (16, 44) (Fig. 4). HDL-FC to LDL transfer increased linearly with HDL-mol% FC (or HDL-FC content), a component of HDL-FC bioavailability (Equation 1; Fig. 5A, B).

### HDL-FC influx into macrophages

As with the HDL-FC transfer to LDL, transfer to macrophages was also a positive function of HDL-FC whether expressed as HDL-mol% FC or HDL-FC content/mg protein (Fig. 5C, D). Although variation for this assay was greater than for transfer to LDL, as indicated by the SD error bars, the linearity and correlations were good, *r*^2^ = 0.71 and 0.92 respectively. Desorption is the rate-limiting step for FC transfer from HDL, so we expected that HDL-FC transfer to LDL and influx into macrophages would correlate. This is not a foregone conclusion, but our data show a good correlation between HDL-FC transfer to LDL and HDL-FC influx into macrophages (Fig. 6; *r*^2^ = 0.82). This correlation has meaningful ramifications. HDL-FC influx and efflux assays are tedious, time-consuming, and require a high level of skill. Moreover, as our data shows, the correlation coefficient for HDL-FC transfer to LDL is marginally higher than that for HDL-FC influx into macrophages. Expressed as HDL-mol%, *r*^2^ = 0.77 for transfer to LDL versus 0.71 for transfer to macrophages; expressed HDL-FC, *r*^2^ = 0.96 for transfer to LDL versus 0.92 for transfer to macrophages. These differences derive, in part, from the difficulty in controlling macrophage cell number, which is quantified as cell protein, compared to LDL concentration, which can be more readily controlled and analyzed with great accuracy.

Several studies have shown that ASCVD prevalence inversely correlates with the magnitude of in vitro macrophage-FC efflux to patient HDL(14–16). One interpretation is that this is a surrogate metric for the in vivo transfer of FC from macrophages within the subendothelial space of the arterial wall, which lowers the atherogenic burden. Conversely, we hypothesized that the reverse process, transfer of HDL-FC (influx) from plasma to the macrophages in the subendothelial space is a metric for ASCVD that is driven by a state of high plasma HDL FCBI with underlying excess particle numbers of HDL that is FC-rich. Plasma HDL-FC and CE are not routinely measured because there is little compelling data connecting one or the other to ASCVD in a way that is significant and clinically meaningful. Moreover, there are few validated mechanistic links between plasma and HDL-FC and ASCVD. Even in the context of robust FC efflux to HDL, the reverse process, FC influx, may be atherogenic and compete with the beneficial effects of FC efflux, especially in cases of high HDL-FC. Although not analyzed in detail, even the Framingham study showed an increase in ASCVD as HDL-C rose to 75 mg/dl (45). A limitation of our study is that FC efflux was not measured so we could not determine whether there was net FC influx to cells. Also, the study was conducted using the J774 mouse macrophage cell line, and the results might be different with human monocyte-macrophages. FC transfer and its balance between cells and plasma in vivo are also likely different.

Our hypotheses on the relationship between HDL-FC and ASCVD were driven mostly by mouse data. Compared to wild-type mice, HDL-FC mol% and HDL concentrations are higher among Scarb1^−/−^ mice, and it is relevant that the multiple pathologies among Scarb1^−/−^ mice are corrected by reducing plasma HDL concentrations and HDL-FC mol% by genetic means, ie, delivery of serum opacity factor by an adeno-associated virus, (26, 27) and by pharmacologic methods, ie, probucol. (29) These pathologies include female infertility (27, 28), erythrocyte defects (26), and ASCVD (29). The goal of the **Houston Heart Study** is to validate the hypothesis that a high plasma HDL-FCBI is associated with excess human ASCVD. If validated, tests of HDL-FC and HDL-P could be used to identify patients requiring HDL-lowering therapies and provoke searches for other diagnostics and tests of HDL-lowering therapies. One of these is probucol, which is currently not approved in the United States, in most part because it lowers HDL-C. Low HDL-C alone versus low HDL-C in the context of metabolic syndrome is not as strong an ASCVD predictor (16). We hypothesize that HDL-FC is a risk factor for ASCVD. Our assays provide two approaches that could reveal if similar correlations exist in humans as have been found in mouse models. One assay, HDL-FC transfer to macrophages, is physiologically meaningful but technically challenging. The other assay, HDL-FC transfer to LDL, is a surrogate that correlates with HDL-FC transfer to macrophages and has the advantages of simplicity, accuracy, and low technical burden. Thus, we anticipate that if other studies in humans confirm our findings, the HDL-FC to LDL assay could be used to determine if the bioavailability of HDL-FC is associated with ASCVD, infertility, and other pathologies associated with high HDL concentrations.

## Data availability

All data are contained within the article and the supplemental data.

## Supplemental data

This article contains supplemental Fig S1–S3 and Tables S1–S6.

## Conflict of interest

The authors declare that they have no conflicts of interest within this article.

## Figures and Tables

**Supplemental Figure S3:**