# Supplementary material for: HDL-free cholesterol influx into macrophages and transfer to LDL correlate with HDL-free cholesterol content
Source: J Lipid Res. 2024 Nov 19;66(1):100707. doi: 10.1016/j.jlr.2024.100707 (PMC11696839; doi:10.1016/j.jlr.2024.100707)
Supplement: Supplemental Table S1 [file mmc1.docx]

| **Supplemental Table S1: Protein and Lipid Assay-Reproducibility** | | | | | | | |
| --- | --- | --- | --- | --- | --- | --- | --- |
|  |  |  |  |  |  | |  |
| Analyte |  | Within-day %CV | |  | Day-to-day %CV | | |
|  |  | mean | SD |  | mean | SD | |
|  |  |  |  |  |  |  | |
| Protein |  | **5.49** | 1.02 |  | **4.01** | 3.69 | |
| Total cholesterol | | **2.98** | 1.72 |  | **3.73** | 3.38 | |
| Free cholesterol | | **2.92** | 1.52 |  | **4.01** | 1.04 | |
| CE |  | **4.97** | 1.65 |  | **4.52** | 3.98 | |
| PL |  | **5.00** | 3.79 |  | **6.69** | 5.13 | |
| TG |  | **4.11** | 0.70 |  | **7.75** | 4.18 | |

Lipoprotein composition was determined using the BioRad DC protein assay kit and the WAKO/Fuji Lipid Assay Kits for PL, Total Cholesterol, Free Cholesterol and Triglycerides. 10 HDL and 10 LDL samples were assayed for each analyte, n=20. Within-day variability is the average %CV for 2 different size aliquots, each assayed in triplicate (n=6 for each sample). Day-to-day %CV is the average for two independent assays done on different days by two research staff.
