# Supplementary material for: HDL-free cholesterol influx into macrophages and transfer to LDL correlate with HDL-free cholesterol content
Source: J Lipid Res. 2024 Nov 19;66(1):100707. doi: 10.1016/j.jlr.2024.100707 (PMC11696839; doi:10.1016/j.jlr.2024.100707)
Supplement: Supplemental Table S3 [file mmc3.docx]

**Supplemental Table S3: Purified HDL and LDL Compositions for Ten Donors
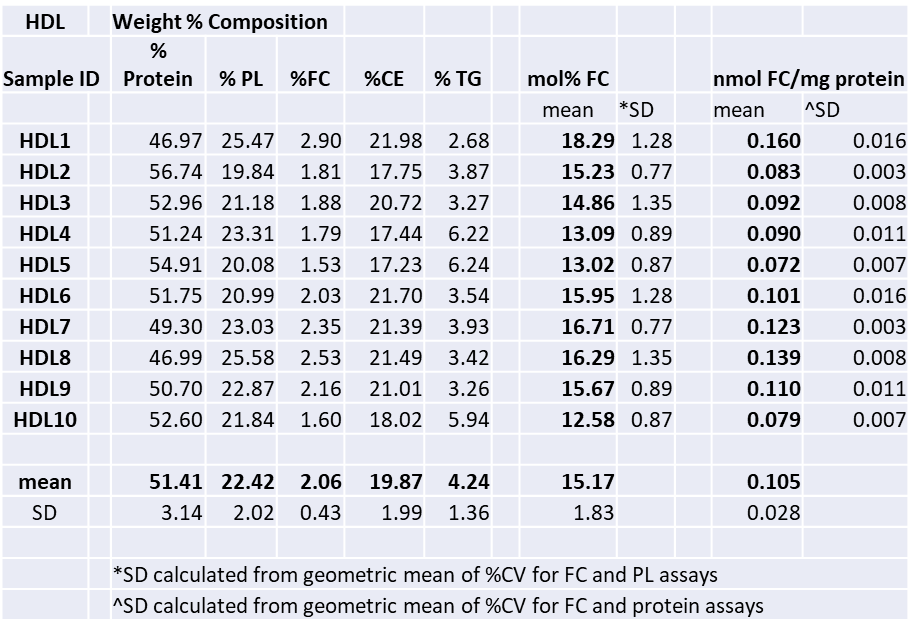

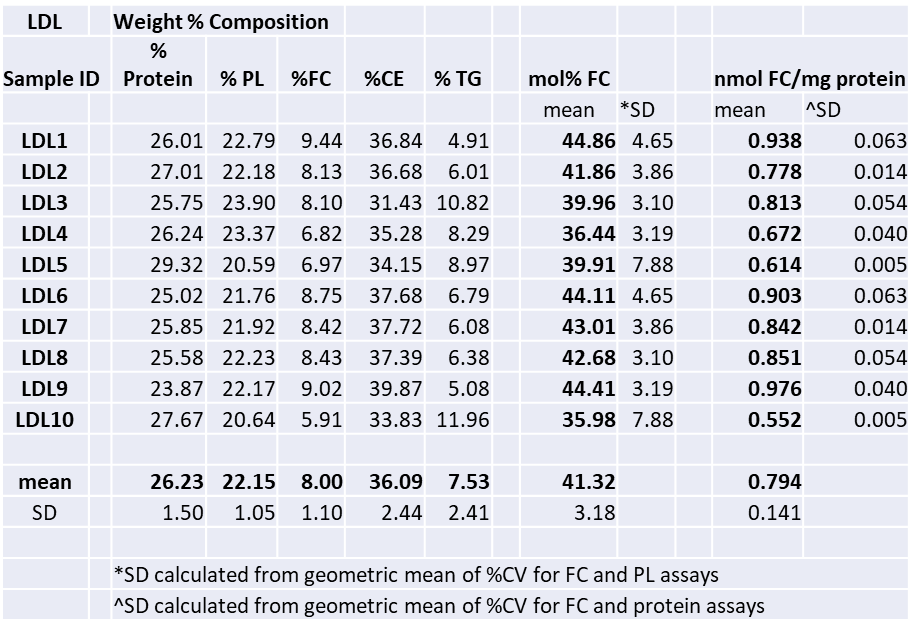
**

HDL and LDL compositions ten donors are in the normolipidemic range.
