# Supplementary material for: HDL-free cholesterol influx into macrophages and transfer to LDL correlate with HDL-free cholesterol content
Source: J Lipid Res. 2024 Nov 19;66(1):100707. doi: 10.1016/j.jlr.2024.100707 (PMC11696839; doi:10.1016/j.jlr.2024.100707)
Supplement: Supplemental Table S4 [file mmc4.docx]

**Supplemental Table S4: Reproducibility of the HDL-[^3^FC] to LDL Transfer Assay**

Preliminary work: Assayed 10 lots of human HDL to determine the variation in transfer to 10 different LDLs as well as a pool made from 5 LDLs. This determined the variability of LDL lots as acceptors of FC from HDL. Determined that nsd among various normolipidemic LDL as acceptors, so will create LDL Pools of several plasma to use for the transfer assays.

Determination of within-day reproducibility and day-to-day reproducibility for two sets of Transfer Experiments, total of 50 and 60 assays of each HDL vs 5 to 6 LDLs done twice 2 to 6 days apart, in quadruplicate. Values are the dpm transferred from donor HDL-[^3^H]FC to acceptor LDL.

1. **Within-day reproducibility: mean %CV for n=3 or 4 replicates**
   1. Data for HDL 1 to 5 vs LDL 1 to 5: mean %CV= **2.48** + 1.16, n=50
   2. Data for HDL 6 to 10 vs LDL 6 to 10 and Pool1: mean %CV = **2.34** + 1.12, n=60
   3. average %CV for all the data, mean %CV= **2.41** + 0.35, n=110

**B. Day-to-day reproducibility of dpm transferred to LDL:**

- 1. Data for HDL 1 to 5 vs LDL 1 to 5: mean %CV= **2.57** + 1.72, n=25
  2. Data for HDL 6 to 10 vs LDL 6 to 10 and Pool1: mean %CV = **1.06** + 1.14, n=30
  3. average %CV for all the data, mean %CV= **1.81** + 1.07, n=55
     1. **Reproducibility over time: ^3^H-FC-HDL transfer to LDL Pool 1 tested again after 10 week interval, stocks stored at 4^o^C:**

HDL6 vs LDL Pool 1 at t_o_: 21.64 + 0.26 pmol FC transferred p=0.388, nsd

HDL 6 vs LDL Pool 1 10 weeks later: 21.41 + 0.42 pmol FC transferred

HDL7 vs LDL Pool 1 at t_o_: 26.50 + 0.23 pmol FC transferred p=0.843, nsd

HDL7 vs LDL Pool 1 10 weeks later: 26.60 + 0.94 pmol FC transferred
