# Supplementary material for: HDL-free cholesterol influx into macrophages and transfer to LDL correlate with HDL-free cholesterol content
Source: J Lipid Res. 2024 Nov 19;66(1):100707. doi: 10.1016/j.jlr.2024.100707 (PMC11696839; doi:10.1016/j.jlr.2024.100707)
Supplement: Supplemental Table S5 [file mmc5.docx]

| **Supplemental Table 5: HDL-FC transfer to LDL differs among HDL but not LDL** | | | | | | | | | | | | | | |  |  |
| --- | --- | --- | --- | --- | --- | --- | --- | --- | --- | --- | --- | --- | --- | --- | --- | --- |
| Data for HDL 1 to 5 to LDL 1 to 5 (Pool A) | | | | | | |  |  |  |  |  |  |  |  |  |  |
|  | HDL-FC | HDL-mol%FC | | **pmol FC transferred** | | | |  |  |  |  |  |  |  |  |  |
|  | pmol/250 ug protein | | | LDL1 |  | LDL2 |  | LDL3 |  | LDL4 |  | LDL5 |  | mean of all 5 LDLs | | |
|  |  |  |  | **mean** | SD | **mean** | SD | **mean** | SD | **mean** | SD | **mean** | SD | **mean** | SD | **%CV** |
| HDL-1 | 39.93 | 18.29 |  | **30.20** | 0.21 | **30.05** | 0.61 | **30.66** | 1.58 | **29.59** | 0.58 | **29.52** | 0.94 | **30.01** | 0.47 | *1.56* |
| HDL-2 | 20.66 | 15.23 |  | **17.11** | 0.39 | **17.09** | 0.08 | **17.13** | 0.19 | **16.90** | 0.48 | **16.79** | 0.42 | **17.00** | 0.15 | *0.88* |
| HDL-3 | 22.95 | 14.86 |  | **19.49** | 0.19 | **19.44** | 0.14 | **19.78** | 1.04 | **19.24** | 0.33 | **19.21** | 0.37 | **19.43** | 0.23 | *1.18* |
| HDL-4 | 22.53 | 13.09 |  | **18.15** | 0.91 | **18.09** | 0.63 | **18.37** | 1.37 | **17.91** | 0.47 | **17.88** | 0.30 | **18.08** | 0.20 | *1.11* |
| HDL-5 | 18.00 | 13.02 |  | **14.58** | 0.28 | **14.54** | 0.18 | **14.68** | 0.65 | **14.39** | 0.22 | **14.33** | 0.27 | **14.50** | 0.14 | *0.97* |
|  |  |  |  |  |  |  |  |  |  |  |  |  |  |  | mean | *1.14* |
|  |  |  |  |  |  |  |  |  |  |  |  |  |  |  |  |  |
| Data for HDL 6 to 10 to LDL 6 to 10 (Pool B) | | | | | | | |  |  |  |  |  |  |  |  |  |
|  | HDL-FC | HDL-mol%FC | | **pmol FC transferred** | | | |  |  |  |  |  |  |  |  |  |
|  | pmol/250 ug protein | | | LDL6 |  | LDL7 |  | LDL8 |  | LDL9 |  | LDL10 |  | mean of all 5 LDLs | | **%CV** |
|  |  |  |  | **mean** | SD | **mean** | SD | **mean** | SD | **mean** | SD | **mean** | SD | **mean** | SD |  |
| HDL-6 | 25.32 | **15.95** |  | **21.66** | 1.02 | **21.73** | 0.51 | **21.64** | 0.61 | **21.95** | 0.37 | **20.99** | 0.56 | **21.59** | 0.36 | *1.67* |
| HDL-7 | 30.82 | **16.71** |  | **26.66** | 1.13 | **26.71** | 0.64 | **26.62** | 0.22 | **26.86** | 0.29 | **25.81** | 0.35 | **26.53** | 0.41 | *1.55* |
| HDL-8 | 34.84 | **16.29** |  | **29.43** | 1.16 | **29.33** | 0.16 | **29.26** | 0.69 | **29.65** | 0.18 | **28.24** | 0.30 | **29.18** | 0.54 | *1.86* |
| HDL-9 | 27.57 | **15.67** |  | **23.58** | 0.79 | **23.58** | 0.53 | **23.55** | 0.67 | **23.80** | 0.24 | **22.74** | 0.03 | **23.45** | 0.41 | *1.76* |
| HDL-10 | 19.67 | **12.58** |  | **17.07** | 0.95 | **17.11** | 0.66 | **17.11** | 0.76 | **17.26** | 0.50 | **16.67** | 0.83 | **17.04** | 0.22 | *1.29* |
|  |  |  |  |  |  |  |  |  |  |  |  |  |  |  | Mean | *1.63* |
