# Supplementary material for: HDL-free cholesterol influx into macrophages and transfer to LDL correlate with HDL-free cholesterol content
Source: J Lipid Res. 2024 Nov 19;66(1):100707. doi: 10.1016/j.jlr.2024.100707 (PMC11696839; doi:10.1016/j.jlr.2024.100707)
Supplement: Supplemental Table S6 [file mmc6.docx]

**Supplemental Table S6: Reproducibility of the Assay for HDL-FC Influx into Macrophages**

**A. Within-day reproducibility: samples assayed in quadruplicate, n= 20 assays**

Set 1 (HDL 1 to 5) within-day %CV, mean + SD: 4.3 + 1.8

Set 2 (HDL 6 to 10) within-day %CV, mean + SD: 4.8 + 1.8

For all 10 HDL, with-in day %CV, mean + SD: 4.6 + 1.8

**B. Day-to-day variability: Each HDL assayed on four different days over two weeks:**

Set 1 (HDL 1 to 5) day-to-day %CV, mean + SD: 10.7 + 1.8

Set 2 (HDL 6 to 10) day-to-day %CV, mean + SD: 8.1 + 0.5

For all 10 HDL, day-to-day %CV, mean + SD: 9.4 + 1.9
